# Novel Three-Dimensional Body Scan Anthropometry versus MR-Pelvimetry for Vaginal Breech Delivery Assessment

**DOI:** 10.3390/jcm12196181

**Published:** 2023-09-25

**Authors:** Anne Dathan-Stumpf, Massimiliano Lia, Christof Meigen, Karoline Bornmann, Mireille Martin, Manuela Aßmann, Wieland Kiess, Holger Stepan

**Affiliations:** 1 Department of Obstetrics, University Hospital Leipzig, 04103 Leipzig, Germany; massimiliano.lia@medizin.uni-leipzig.de (M.L.); karoline.bornmann@medizin.uni-leipzig.de (K.B.); holger.stepan@medizin.uni-leipzig.de (H.S.); 2 LIFE Leipzig Research Center for Civilization Diseases, University of Leipzig, 04103 Leipzig, Germany; christof.meigen@medizin.uni-leipzig.de (C.M.); manuela.assmann@medizin.uni-leipzig.de (M.A.); wieland.kiess@medizin.uni-leipzig.de (W.K.); 3 Department of Diagnostic and Interventional Radiology, University Hospital Leipzig, 04103 Leipzig, Germany; mireille.martin@medizin.uni-leipzig.de; 4 Department of Pediatrics, University Hospital Leipzig, 04103 Leipzig, Germany

**Keywords:** breech delivery, birth planning, MR-pelvimetry, three-dimensional pelvimetry, body scan, obstetric conjugate

## Abstract

In this prospective, monocentric study, we investigated the potency of a novel three-dimensional (3D) body scanner for external pelvic assessment in birth planning for intended vaginal breech delivery. Between April 2021 and June 2022, 73 singleton pregnancies with intended vaginal birth from breech presentation (>36.0 weeks of gestation) were measured using a pelvimeter by Martin, a three-dimensional body scanner, and MR-pelvimetry. Measures were related to vaginal birth and intrapartum cesarean section. A total of 26 outer pelvic dimensions and 7 inner pelvic measurements were determined. The rate of successful vaginal breech delivery was 56.9%. The AUC (area under the curve) of the obstetric conjugate (OC) measured by MRI for predicting the primary outcome was 0.62 (OR 0.63; *p* = 0.22), adjusted for neonatal birth weight 0.66 (OR 0.60; *p* = 0.19). Of the 22 measured 3D body scanner values, the ratio of waist girth to maternal height showed the best prediction (AUC = 0.71; OR 1.27; *p* = 0.015). The best predictive pelvimeter value was the distantia spinarum with an AUC of 0.65 (OR = 0.80). The 3D body scanner technique is at least equal to predict successful vaginal breech delivery compared to MRI diagnostics. Further large-scale, prospective studies are needed to verify these results.

## 1. Introduction

The prevalence of fetal breech presentation around term is reported at 2–4% [1,2]. Since the Term Breech Trial, which showed significantly higher perinatal morbidity and mortality after vaginal birth [3], the planned cesarean section has been practiced internationally as the preferred mode of delivery [4], even though numerous studies have disproved the results of the Term Breech Trial [5,6,7] and the study itself was withdrawn due to methodological errors.

In Germany, a number of perinatal centers practice vaginal delivery from breech presentation. Although birth planning currently includes MR-pelvimetry in primiparous women with measurement of the obstetric conjugate (target > 12.0 cm) to detect a feto-pelvic disproportion, the role of MR-pelvimetry is unclear. Compared to women with a successful vaginal delivery, those with a cesarean section more frequently showed a lower value of the obstetric conjugate (OC) [8,9,10,11] and interspinous distance (ISD) [12]. Although some studies have shown that a higher OC is a predictive value for a successful vaginal birth from breech presentation, the predictive value of this parameter is conflicting.

Before MRI pelvimetry was established to determine the inner pelvic distances, efforts were made to determine a possible feto-pelvic disproportion by measuring the outer pelvis. As a tool for pelvic measurement, the pelvimeter was used. The external conjugate minus 9 cm was used as an approximation for the Obstetric conjugate [13]. Although there are hardly studies on measuring the outer pelvis using the pelvimeter in literature, the determination of a single outer pelvic value seems to hardly allow any conclusions about a feto-pelvic disproportion [14].

Recently, the anthropometric measurement of the outer pelvis was investigated using three-dimensional (3D) camera technology to detect such a disproportion [15,16]. Especially in developing countries with poor infrastructural conditions and long distances to the next maternity hospital, it could be shown that this inexpensive, fast, and portable technology offers new possibilities in measuring pelvic anatomy and predicting a feto-pelvic imbalance. Compared to conventional manual anthropometry, measurement errors and inherent fluctuations can be significantly reduced and new parameters established [16]. 

Since 2011, as part of the LIFE-Child study [17,18], pregnant women have been measured three-dimensionally using eye-safe laser technology in a double-triangulation process [19]. This method offers the possibility to determine numerous defined body circumferences, distances, and ratios automatically and non-invasively within a few seconds.

So far, there are no studies on the 3D body scan in pregnant women or the correlation between three-dimensional external pelvic measurements and MR-pelvimetry. The aim of the study was to investigate whether outer measurements of the pelvis, using the pelvimeter and 3D body scanner, are related to MRI measures. Secondly, we wanted to answer the question of whether 3D body scan measurements are superior to MR-pelvimetry and can be used to predict a successful vaginal breech delivery.

## 2. Materials and Methods

### 2.1. Study Population and Design

We conducted a prospective single-center study in a purely Caucasian, Central European cohort. Between April 2021 and June 2022, mainly primiparous women with a singleton pregnancy and persistent breech presentation at >36.0 weeks of gestation were recruited for the study during their birth planning visit at the University Hospital Leipzig. Patients with a medical indication for a planned cesarean section (severe fetal malformations, gestational age ≤ 36.0 weeks of pregnancy, estimated birth weight below 2500 g or above 4000 g) were also excluded.

After recruitment, the three examination methods of inner (MR-pelvimetry) and outer pelvic measuring (pelvimeter, 3D body scan) were carried out with a maximum interval of 5 days. 

In addition to the anthropometric examinations of the pelvis, the following data were recorded: Maternal age, BMI at the time of the MR-pelvimetry, gestational age at the time of the MRI examination, parity, delivery mode, need for maneuvers in a vaginal delivery, reason for cesarean, gestational age at delivery, pH-value, APGAR score after 1/5/10 min, and neonatal birth weight and sex. Information on maternal height and weight before pregnancy was taken from the maternity booklet. 

Studies on humans are carried out in accordance with the Declaration of Helsinki (1975). The study was approved by the Ethical Committee of the University of Leipzig (reference number: 086/21-ek; IRB00001750, date of approval: 16 March 2021). Additionally, appropriate written informed consent was obtained from all patients included in this study.

### 2.2. Pelvimeter

A pelvimeter by Martin was used for manual outer pelvimetry. The following measurements were recorded: *Distantia spinarum* (distance between the two spinae iliacae anteriores superiores), *Distantia cristarum* (farthest distance between the two cristae iliacae), *Distantia trochanterica* (distance between the two greater trochanters), and *External Conjugate* (distance from the upper edge of the symphysis to the processus spinosus of the 5th lumbal vertebra, corresponds to the top point of the Michaelic rhombus). To minimize interpersonal measurement errors, the measurement of the pelvis with the pelvimeter was exclusively carried out by two midwives.

### 2.3. Magnetic Resonance Imaging (MR) Pelvimetry

At the University Hospital Leipzig, MR-pelvimetry is part of the standard clinical procedure in planning delivery from breech presentation. The primary target parameter is the Obstetric conjugate (OC, sagittal distance between the dorsal edge of the promontorium and the dorsal surface of the symphysis, target > 12.0 cm). Secondary target measures were pelvic width (PW, sagittal distance between the dorsal surface of the pubic symphysis and the middle of the 3rd sacral vertebrae), sacral pelvic outlet diameter (SOD, sagittal distance between the inferior border of the pubic symphysis and sacroiliac joint), coccygeal-pelvic outlet (CPO, sagittal distance from the coccyx tip to the inferior border of the pubic symphysis), interspinous distance (ISD, distance between the sciatic spines), and intertuberous distance (ITD, distance between the two sciatic tubes) [12]. 

The MRI examination takes place between the 36th and 38th weeks of gestation (wog) using a 1.5-T-MRI system (Symphonie, Siemens Healthcare, Erlangen, Germany) in the supine position. A T2-weighted single-shot turbo spin-echo sequence (HASTE) is used for the sagittal section, and a T1-weighted spin-echo sequence (SE) is used for the axial section. The slice thickness in both sequences is 5 mm. Special patient preparation and administration of contrast media are not necessary.

The evaluation of the MR-pelvimetry was carried out by two senior physicians from the Department of Diagnostic and Interventional Radiology, University Hospital Leipzig. 

### 2.4. Three-Dimensional (3D) Body Scan

The 3D body scan anthropometry was performed with the Vitus Smart 3D scanner from Vitronic [19]. Using eye-safe laser technology, three-dimensional images are designed within a few seconds in a double-triangulation process. The images are generated in AnthroScan 2.9.9 software, which, like the scanner itself, conforms to the international standard ISO 20685 [20]. The images can also be edited in the software. Measurement planes can be corrected and supplemented as required.

With a resolution of 1 mm, an average of 350,000 points are recorded per scan. A total of 22 potentially relevant body scanner values were analyzed. The exact designation and schematic representation of the automatically generated body scanner measurement values are shown in Table S1. In addition, it is possible to define certain points even more precisely using marking points. For a better evaluation of the measurement planes and comparability of the body scanner measurements with the pelvimeter, the following bone structures were marked with validated “marking points” from the Institute for Applied Training Sciences Leipzig (IAT): Both spinae iliacae anteriores superiores, the highest points of the cristae iliacae, trochanteres majores, the midpoint of the upper edge of the symphysis, and the 5th lumbal vertebra (upper point of Michaelic rhombus). The marking points (diameter of approximately 1 × 1 cm) were glued to the skin over the corresponding bone structures. The marking of the bone structures and measurement in the body scanner were primarily carried out by two employees in order to keep interpersonal bias to a minimum. The values of the external conjugate, distantia spinarum, distantia intercristarum, distantia trochanterica, and the distance crista to trochanter major on the right side were generated as additional variables and subsequently measured with the help of the marking points. There is a deviation in case numbers of the body scanner data, since three automatically generated datasets were missing values, while the additional measurements generated via the marking points could be measured.

**Figure 1:**
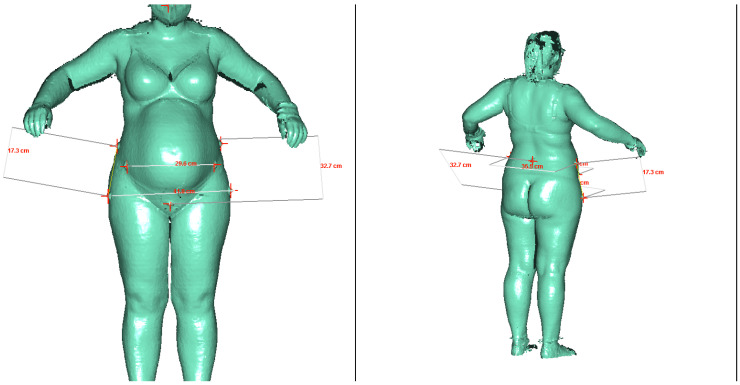
Example images of a measurement in the 3D body scanner from the front and side rear view.

The measurement in the body scanner was carried out while standing in an upright position with a foot closure approximately 5–10 cm open. The arms were spread out to the sides of the body (Figure 1). For the measurement, the pregnant woman only wore light-colored, tight-fitting underwear.

The measurement in the body scanner was carried out while standing in an upright position with a slightly open footrest. The arms were spread out to the sides of the body. The red crosses are located in the center of the additionally glued marking points above the bony structures. Thus, the additional measurement values of the external conjugate, distantia spinarum, distantia intercristarum, distantia trochanterica, and the distance crista to trochanter major (right) could be determined.

### 2.5. Statistical Analysis

The main dependent variable and primary outcome was intrapartum cesarean section. The independent variables were selected based on the results of previous literature [8,9,12] and included all measures performed by the body scanner, as it was the main aim of the study to explore its clinical value. Different logistic regression models were built for the prediction of intrapartum cesarean section in our cohort. A descriptive analysis of the independent variables was performed and is presented in Table 1 and Table 2. Continuous variables are presented as medians with interquartile ranges and categorical variables are presented as frequencies (%). The Mann–Whitney U test was used to compare continuous variables and the chi-square test for categorical variables. Univariable analysis was used to identify independent variables with an association with the primary outcome and were considered for inclusion in the multivariable logistic models. Since the number of the whole cohort and the events (intrapartum cesarean section) were limited, a maximum of two variables were included in these logistic models. Due to multiple model tests, adjusted *p*-values according to the false discovery rate by Benjamini–Hochberg were additionally indicated. Odds ratios (ORs) were obtained from the logistic regression analysis. The diagnostic accuracy of each model was assessed through sensitivity, specificity, receiver-operating characteristics (ROC) curves, and the area under the ROC curve (AUC) with a 95% CI. Paired ROC curves were compared by the DeLong method, and the reference was the ROC of the OC [21].

The statistical software package R (Version 4.1.0) [22] and the IBM Statistical Package for Social Sciences (IBM SPSS V.27) were used for data analysis and creation of graphics.

## 3. Results

### 3.1. Study Population

**Figure 2:**
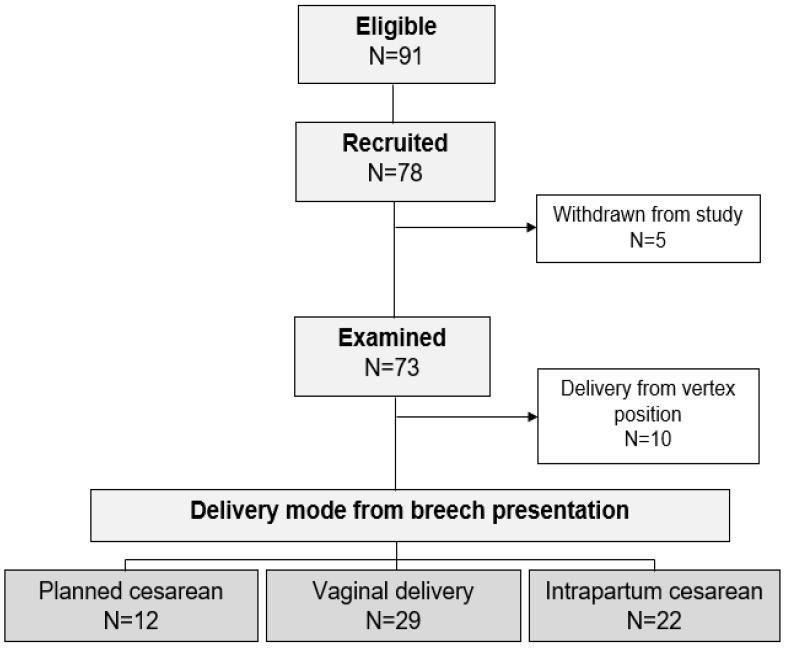
Presentation of the study cohort.

A total of 78 patients with a singleton pregnancy in breech presentation were recruited during the study period. Five of the included patients subsequently withdrew their consent or decided to undergo a planned cesarean section. After excluding 10 pregnant women where the fetus turned spontaneously into a vertex position after the 36th week of gestation and 12 planned cesareans (partly giving birth in external hospitals), a total of 51 patients were finally included in the analysis (Figure 2). The percentage of successful vaginal births from the breech presentation was 56.9%. Table 1 summarizes the examination characteristics of the total cohort. 

The recruitment rate during the study period was almost 86% (78/91). After excluding deliveries from vertex positions and planned cesareans, a total of 51 patients were included in the analysis.

A total of five women had cesarean sections in previous pregnancies (one woman had two previous cesarean sections). Since these women have never given birth vaginally, they are treated as nulliparous according to hospital standards and undergo normal birth planning with MRI pelvimetry. 

All women with an OC < 12.0 cm decided on planned cesarean section. The leading reason for an intrapartum cesarean section was birth arrest (22.9%). One patient who appeared suitable for vaginal delivery at the time of birth planning showed a macrosomic fetus estimated over 4000 g during labor, therefore a cesarean section was indicated.

The mean gestational age at delivery was 39.4 wog. All children were born at term (>37.0 wog). On average, the measurements of the outer and inner pelvis were carried out in the 36.5 wog. With regard to the time of the outer and internal pelvic measurement, time of delivery, and neonatal birth weight, the two groups of successful vaginal breech delivery (N = 29) and intrapartum cesarean section (N = 22) were completely comparable. Also, the rate of epidural anesthesia did not differ significantly between the two delivery modes (vaginal delivery 62.1% (18/29), intrapartum cesarean section 59.1% (13/22), *p* = 0.83). The rate of obstetric maneuvers was 44.8% (13/29). As described before [23], neonates after intrapartum cesarean section showed significantly better pH values, but no differences could be found with regard to the 5 min APGAR scores (Table 2).

**Table 2:** Results of the outer and inner pelvic measurement.

Variables		Vaginal Delivery from Breech					Intrapartum (Secondary) Cesarean					*p*-Value	adj. *p*-Value
		N	Mean	SD	Median	95% CI	N	Mean		Median	95% CI		
	gestational age at pelvimetry [weeks]	29	36.6	0.8	37.1	36.5; 37.1	22	36.6	0.5	37.0	36.5; 37.0	0.85	0.88
	BMI [kg/m²] before pregnancy	25	21.9	2.9	21.4	20.8; 23.0	22	23.1	3.4	22.2	21.6; 24.6	0.14	0.58
	BMI [kg/m²] at diagnostics	25	26.7	3.3	25.8	25.4; 28.1	22	28.7	3.9	28.6	27.1; 30.4	0.03	0.41
	BMI gain [kg/m²]	25	4.6	1.2	4.6	4.1; 5.1	22	5.7	1.7	5.8	4.9; 6.4	<0.001	0.04
**MR pelvimetry**	obstetrical conjugate [cm]	29	13.1	0.7	12.9	12.86; 13.36	22	12.8	0.9	12.8	12.45; 13.25	0.15	0.57
	pelvic width [cm]	29	13.8	0.9	13.9	13.49; 14.20	22	13.5	0.9	13.3	13.12; 13.92	0.28	0.55
	pelvic constriction [cm]	29	11.7	1.1	11.7	11.28; 12.13	22	12.0	0.8	12.1	11.65; 12.33	0.36	0.59
	sacral pelvic outlet diameter [cm]	29	13.6	0.7	13.7	13.36; 13.87	22	13.4	0.9	13.4	12.99; 13.79	0.31	0.56
	coccygeal-pelvic outlet [cm]	29	8.6	1.1	8.6	8.15; 8.96	22	8.7	0.9	8.7	8.32; 9.10	0.99	0.99
	interspinous distance [cm]	29	11.2	0.7	11.3	10.91; 11.47	22	10.9	1.0	11.0	10.46; 11. 31	0.13	0.58
	intertuberous distance [cm]	29	14.3	1.1	14.2	13.93; 14.74	22	13.9	1.4	14.2	13.23; 14.51	0.36	0.57
**Pelvimeter**	external conjugate [cm]	29	23.9	2.2	24.0	23.01; 24.71	21	23.6	2.3	24.0	22.52; 24.61	0.76	0.92
	distantia spinarum [cm]	29	24.3	1.8	25.0	23.67; 25.02	21	23.7	1.6	24.0	22.98; 24.43	0.07	0.61
	distantia intercristarum [cm]	29	27.8	1.6	28.0	27.15; 28.37	21	28.0	2.5	27.5	26.83; 29.10	0.86	0.63
	distantia trochanterica [cm]	29	33.2	2.3	33.0	32.28; 34.06	20	33.1	2.6	32.1	31.85; 34.30	0.58	0.79
**3D bodyscan**	waist to high hip back (5070) [cm]	25	7.7	1.5	7.6	1.06; 8.32	22	7.1	1.5	7.4	6.47; 7.83	0.25	0.55
	distance waistband to high hip back (5075) [cm]	25	4.1	2.7	4.0	2.95; 5.15	22	4.2	1.9	4.2	3.33; 4.97	0.89	0.94
	waist to buttock (5080) [cm]	25	21.7	1.6	21.7	21.02; 22.31	22	21.2	1.7	21.1	20.50; 21.97	0.34	0.58
	distance waistband to buttock (5085) [cm]	25	17.3	2.9	17.3	16.12; 18.48	22	17.2	2.0	17.2	16.31; 18.10	0.86	0.93
	scrotch length, rear (6012) [cm]	25	42.9	2.8	42.6	41.74; 44.01	22	43.1	2.6	42.7	41.98; 44.25	0.82	0.96
	scotch length at waistband (6015) [cm]	25	69.9	10.4	70.4	65.65; 74.24	22	72.7	10.1	74.9	68.27; 77.21	0.27	0.56
	waist girth (6510) [cm]	25	98.8	7.1	97.6	95.90; 101.73	22	102.5	6.8	103.7	99.49; 105.51	0.06	0.61
	middle hip (6512) [cm]	25	110.8	9.0	107.9	107.12; 114.56	22	113.8	8.7	114.7	109.99; 117.71	0.19	0.57
	waist band (6520) [cm]	25	103.6	7.2	102.2	100.66; 106.59	22	106.0	7.0	106.4	102.86; 109.11	0.23	0.53
	waist to buttock hight right (7011) [cm]	25	21.4	1.6	20.9	20.71; 22.10	22	20.6	1.6	20.8	19.93; 21.34	0.19	0.65
	waistband to buttock hight right (7016) [cm]	25	15.0	3.5	16.0	13.54; 16.43	22	15.2	2.8	15.4	13.93; 16.42	0.90	0.92
	waist to hip right (7021) [cm]	25	36.1	2.0	36.0	35.26; 36.90	22	35.5	1.9	35.3	34.63; 36.31	0.31	0.57
	high hip girth (7510) [cm]	25	109.4	7.8	107.3	106.16; 112.60	22	112.2	8.2	112.7	108.61; 115.87	0.21	0.51
	buttock girth (7520) [cm]	25	109.4	9.5	107.2	105.51; 113.38	22	110.9	9.5	112.0	106.68; 115.13	0.52	0.73
	hip girth (7525) [cm]	25	110.5	9.8	108.5	106.42; 114.50	22	112.1	9.4	112.7	107.90; 116.28	0.48	0.70
	belly circumference (7540) [cm]	25	107.8	7.3	105.9	104.79; 110.82	22	110.6	7.8	111.0	107.10; 114.00	0.20	0.56
	maximum belly circumference (7545) [cm]	25	108.7	7.6	106.4	105.61; 111.88	22	111.6	8.0	111.9	108.10; 115.17	0.19	0.60
	external conjugate [cm]	26	29.3	2.5	28.7	28.25; 30.27	22	30.0	2.1	29.9	29.08; 30.98	0.09	0.59
	distantia spinarum [cm]	26	27.5	2.4	27.7	26.53; 28.44	22	27.3	2.1	27.0	26.42; 28.26	0.74	0.92
	distantia intercristarum [cm]	26	33.7	2.7	33.5	32.60; 34.75	22	34.6	2.8	34.8	33.32; 35.82	0.12	0.63
	distantia trochanterica [cm]	26	39.0	3.3	38.6	23.70; 30.35	22	38.9	3.7	38.4	27.25; 40.49	0.72	0.92
	distance crista to trochanter (right) [cm]	26	19.9	3.2	19.4	18.56; 21.16	22	18.7	3.7	17.7	17.08; 20.33	0.09	0.55
**delivery**	gestational age at delivery [weeks]	29	39.4	0.9	39.5	39.1; 39.6	22	39.5	1.2	40.0	39.1; 40.1	0.67	0.88
	birth weight [g]	28	3323.8	385.1	3270.0	3177.3; 3470.3	22	3397.5	262.6	3375.0	3281.1; 3513.9	0.21	0.54
	pH value	28	7.19	0.07	7.19	7.16; 7.22	22	7.25	0.10	7.27	7.21; 7.30	0.005	0.09
	APGAR score 5 min	28	9.2	0.7	9.0	8.9; 9.5	22	9.3	1.1	10.0	8.8; 9.8	0.41	0.62

Shown are the three different methods of inner and outer pelvis measuring, divided by delivery mode. Due to the small number of cases, the medians are given in addition to the mean values. Differences between the two different delivery modes were calculated using the Mann–Whitney U-test. A *p*-value < 0.05 was considered as significant and are shown in bold. Due to multiple model tests, the false discovery rate-adjusted *p*-values according to Benjamini–Hochberg are also given. The numbers in brackets behind the body scanner values correspond to the description from the measurement value catalog (Supplementary Materials). 5′ APGAR-value, Appearance-Pulse-Grimace-Activity-Respiration value 5 min after delivery; 95% CI, 95%-confidence interval; adj. *p*-value, adjusted *p*-value according Benjamini-Hochberg; BMI, Body-Mass-Index; max, maximum; min, minimum; N, number; SD, standard deviation.

### 3.2. Outer and Inner Measurement of the Pelvis and BMI Gain

Table S2 summarizes all of the inner and outer pelvic measurements for the total cohort, including the pregnancies with planned cesarean and vertex presentation. Table 2 shows the same table for the 51 included subjects, subdivided according to the delivery mode in ‘successful vaginal delivery from a breech presentation’ versus ‘intrapartum cesarean section’. No significant difference between the two delivery modes could be shown for any of the measured values in the MR-pelvimetry, the external measurement with the pelvimeter, or the 3D body scan anthropometry (Table 2). A trend could be shown for the 3D anthropometry of the waist girth (*p* = 0.059): Women with successful vaginal delivery had a mean girth of 98.8 cm (median 97.6 cm) compared to women with an intrapartum cesarean section of 102.5 cm (median 103.7 cm). Notably, when waist circumference was related to the woman’s height, this ratio was found to be significantly different between the two subgroups (0.58 ± 0.03 versus 0.61 ± 0.04, *p* = 0.014). 

The two methods for measuring the outer pelvis using the pelvimeter and 3D body scanner showed, despite the additional marking points in the body scanner, large differences. Nevertheless, both methods showed significant dependencies for the external conjugate (*p* = 0.002), distantia spinarum (*p* < 0.001), and distantia trochanterica (*p* < 0.001) in the linear regression analysis, and the adjustment for the maternal BMI at the time of the measurement showed no influence.

The two subgroups of delivery mode differed significantly in terms of BMI gain during pregnancy. While the mean BMI gain in the group of vaginal births was 4.6 kg/m^2^, the women with an intrapartum cesarean gained an average of 5.6 kg/m^2^ (*p* < 0.001, adj. *p* = 0.04).

**Figure 3:**
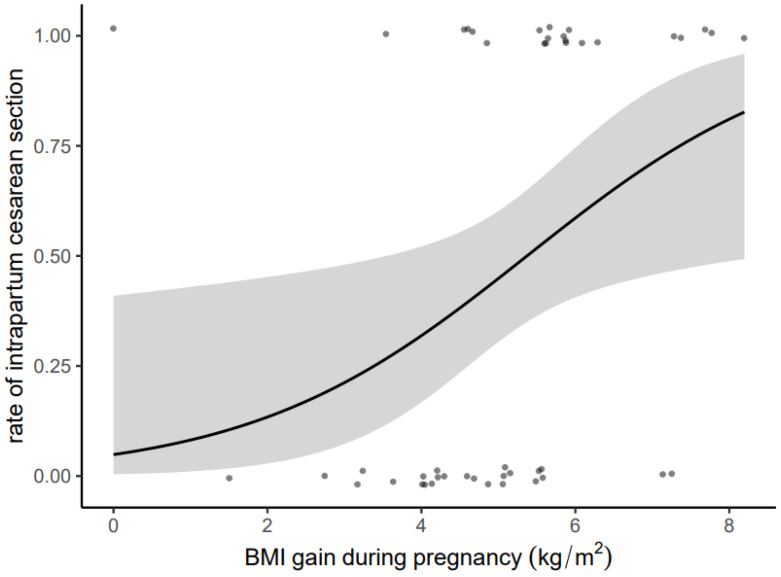
Presentation of the relation between BMI gain during pregnancy (kg/m^2^) and probability for an intrapartum cesarean section by a regression line (black).

Figure 3 shows the relationship between BMI gain during pregnancy (kg/m^2^) and the probability of a cesarean section. The graphic clearly shows a rapid increase in the probability of a cesarean section if the BMI gain is 5 kg/m^2^.

The gray area shows the 95% confidence interval. The points below the regression curve indicate successful vaginal births from a breech presentation, the points above the curve indicate women with an intrapartum cesarean section. Small random variation has been added to the dots in order to avoid overplotting and improve visualization. Women with a successful vaginal delivery from breech presentation showed a significantly lower BMI gain during pregnancy compared to women with intrapartum cesarean (mean 4.6 kg/m^2^ versus 5.7 kg/m^2^, *p* < 0.001). 

### 3.3. Predictive Value of the Different Models

In the prediction of an intrapartum cesarean section, the obstetric conjugate showed an AUC of 0.62 (OR of 0.62, *p* = 0.22). Adjusted for neonatal birth weight the AUC was 0.66 (Table 3). Of the classic pelvic dimensions determined by the pelvimeter, the distantia spinarum showed the best prediction for the primary endpoint (AUC = 0.65, OR 0.80). The historical measure of the external conjugate minus 9 cm [13] showed the worst prediction of all represented models in Table 3 (AUC = 0.53, OR = 0.94). Significance was not found for any of these regression analyses (Table 3). In total, of the 22 analyzed body scanner measurements, the ratio of waist girth to maternal height showed the best discrimination with an AUC of 0.71 (OR 1.27, *p* = 0.015). However, the overall best prediction for the primary endpoint was achieved with the model of BMI gain during pregnancy (AUC = 0.79). With rising BMI gain, there was a significant increase in the odds of a cesarean section by a factor of 1.27 (*p* = 0.026).

**Figure 4:**
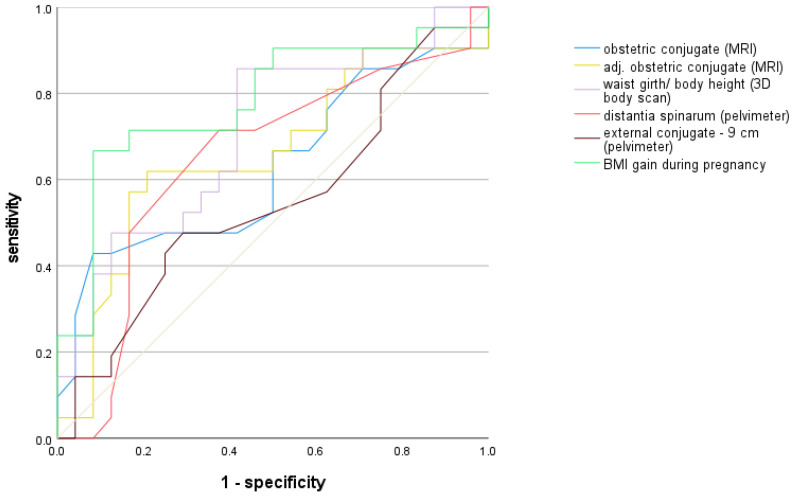
Presentation of the ROC curves with prediction of an intrapartum (secondary) cesarean section.

The models of the best predictive values of the three different examination methods of the inner and outer pelvis as well as the historical measure of the external conjugate minus 9 cm and the obstetric conjugate as a reference are shown as ROC curves in Figure 4.

Shown are the best predictive values of the three different examination methods of the inner and outer pelvis as well as the historical measure of the external conjugate minus 9 cm. The diagonal reference line is shown in grey.

However, in paired comparisons of the ROC curves according to the DeLong method, each with the clinically relevant reference measure of the obstetric conjugate, no significant differences were found (Table 3). 

The prediction models of waist girth (*p* = 0.032) and waist girth to height (*p* = 0.013) showed a significantly better prediction of the primary endpoint compared to the historical measure, while no difference was found between the historical measure and the OC (DeLong 2, Table 3).

## 4. Discussion

This is the first study in pregnant women using both an innovative 3D body scan and MRI to take pelvic measures in women planning vaginal breech delivery. We were able to show that an inexpensive, non-invasive, and fast measurement of the outer pelvis can achieve an at least equivalent prediction of a successful vaginal breech delivery as the standard reference measure of the obstetric conjugate.

Intended vaginal breech delivery requires birth planning and a selection of suitable women. At the University Hospital Leipzig, like others, the nationally and internationally recommended requirements for a vaginal breech birth apply [24,25]. In addition, MR-pelvimetry is performed to determine the obstetric conjugate, as various studies have shown a successful vaginal birth with an OC of ≥12.0 cm [8,9,10]. Nevertheless, the benefit of MR-pelvimetry in the prediction of a successful vaginal delivery is unclear, especially since previous publications do not name any specific prediction values.

Klemt et al. found a significant difference in OC between vaginal breech delivery and intrapartum cesarean section with an aOR of 1.56 per additional cm for a successful vaginal birth [9]. This corresponds to an adjusted OR of 0.64 for the intrapartum cesarean section (1/1.56), which is similar to our findings (OR = 0.63). This is also confirmed by comparing the mean values for the OC between the two delivery modes [9]. Also, Hoffmann et al. were able to show the same mean values for the obstetric conjugates for the two modes of delivery. Their study also found no significance for the OC between vaginal birth and cesarean section in the regression analysis, which also confirms the results of our study [12]. Hoffmann et al. named the ISD, measured in the MR-pelvimetry, with an AUC of 0.67 as a significant predictor of a successful vaginal birth from breech presentation. In our study, the AUC of the ISD was 0.63, although no significance could be demonstrated.

To our knowledge, there is no study that investigates the prediction of successful vaginal breech birth using anthropometric 3D body scan measurements. According to a systematic review, the 3D scanner allows automated, quick, and easy measurements of different body tissues. According to the authors, the measurements appear to be reproducible, reliable, accurate, and correlate with other measurement techniques [26]. The idea of a pelvic assessment without MRI might be an attractive alternative if MRI is not available. Moreover, an anthropometric 3D body scan measurement is able to challenge traditional outer pelvic measures. Interestingly, despite the marking of the bone structures via marking points, there were significant length differences between the same outer pelvic dimensions, measured by the pelvimeter and the body scanner. This results from the fact that the pelvimeter is specifically placed on the bony structures and, thus, changes the skin level. The 3D body scanner only scans the skin surface. Therefore, the measurement of subcutaneous fat tissue was considered during study preparation in order to adjust the results later accordingly. However, due to the different intra-individual and inter-individual distribution of subcutaneous fat, the measurement was removed from the study protocol [27]. Nevertheless, it can be assumed that a comparison of conventional anthropometry (e.g., measurement of waist girth) and body scanner anthropometry would result in reliable, comparable values [26] even in pregnant women.

In summary, the results show the limited prediction of the OC measured in the MRI. We were able to show that equivalent prediction can be achieved by time-saving, cost-effective anthropometric measurements like the waist girth. In our opinion, the strength of MR-pelvimetry is not in predicting birth success from breech presentation. Rather, research suggests that determining the ITD using MRI can predict the duration of the active second stage of labor as well as the rate of obstetrical maneuvers [28]. 

A limitation of this study is certainly the small number of cases. This results partly from the unusually high rate of spontaneous versions into vertex position (13.7%). In the literature, the incidence for a spontaneous version from the 37th week of gestation is given as 6–9%, and in primiparous women, it is as high as 2.3% [29,30]. 

Further large-scale, prospective studies should be carried out to confirm the effect of the BMI gain during pregnancy as well as the influence of waist girth on the success of vaginal breech delivery.

## 5. Conclusions

The usefulness and benefit of measuring the OC with MR-pelvimetry in predicting the success of a vaginal breech delivery is controversial. In our study, we show evidence that anthropometric outer measures can also be obtained by the 3D body scan technique, which may be at least equivalent in predicting an intrapartum cesarean section to measurements of internal pelvic dimensions. Future studies have to show which methods of pelvic assessment are best to forecast successful vaginal breech delivery. 

## 6. Contribution

What are the novel findings of this work?

For the first time, we were able to show that the relation of waist girth to maternal height, measured anthropometrically in an innovative 3D body scanner, is at least equivalent to the prediction to the current standard measure of the obstetric conjugate measured by MR-pelvimetry.

What are the clinical implications of this work?

Anthropometric 3D body scan measurement is able to challenge traditional outer pelvic measures and might be an attractive alternative in birth planning in vaginally intended breech deliveries if MRI is not available. 

## Figures and Tables

**Table 1:** Characteristics of the overall study cohort (N = 73), including the 10 pregnant women where the fetus turned spontaneously into a vertex position after the 36th week of gestation.

Character		N	%
Parity	0	68	93.2
	1	4	5.5
	2	1	1.4
Presentation	vertex	10	13.7
	breech	63	86.3
Delivery mode	vaginal	37	50.7
	planned (primary) cesarean	12	16.4
	Intrapartum (secondary) cesarean	24	32.9
Reason for cesarean	obstetric conjugate < 12.0 cm	12	34.3
	fetal estimated weight < 2500 g or >4000 g	1	2.9
	pathological CTG	3	8.6
	birth arrest	8	22.9
	difficult birth position	3	8.6
	maternal wish	5	14.3
	others	3	8.6

Shown are different study characteristics for the total cohort. Five women had a cesarean section in the previous pregnancy and therefore underwent birth planning with an MRI pelvimetry. Two of ten women with fetus in vertex presentation received an intrapartum cesarean section. In one case, the reason for intrapartum cesarean section (vertex presentation) could not be determined as birth took place in another hospital. N, number.

**Table 3:** Logistic regression model for predicting a secondary cesarean section.

Independent Variable	Regr. Coefficient	95% CI		*p*-Value	OR	AUC	DeLong 1	DeLong 2
obstetric conjugate (MRI) [cm]	−0.47	0.29	1.33	0.22	0.63	0.62	*Ref.*	0.33
adj. obstetric conjugate * [cm]	−0.52	0.28	1.28	0.19	0.60	0.66		
interspinous distance (MRI) [cm]	−0.45	0.32	1.28	0.20	0.64	0.63	0.95	0.21
external conjugate—9 cm [cm]	−0.06	0.73	1.22	0.64	0.94	0.53	0.33	*Ref.*
waist girth (body scan) [cm]	0.08	0.99	1.18	0.08	1.08	0.66	0.92	0.032
waist girth/body height (body scan)	0.24	1.05	1.55	0.015	1.27	0.71	0.35	0.013
adj. external conjugate ^#^ (body scan) [cm]	0.23	0.94	1.67	0.12	1.26	0.67		
distantia spinarum (pelvimeter) [cm]	−0.22	0.57	1.12	0.20	0.80	0.65	0.80	0.15
BMI gain during pregnancy [kg/m^2^]	0.55	1.07	2.83	0.026	1.74	0.79	0.13	0.007

* Adjusted for neonatal birth weight; ^#^ adjusted for maternal hight. Shown are the predictive models for the primary endpoint of an intrapartum cesarean section. Identified independent variables of the three inner and outer pelvic measurements with a *p*-value of <0.15 in the univariate regression analysis were chosen for the multivariate logistic regression analysis. In total, the best predictive model was achieved using the BMI gain. A *p*-value < 0.05 was considered as significant. In prediction of the primary endpoint, all models were equivalent to the OC (DeLong 1). 95% CI, 95%-confidence interval; AUC, area under the curve; OR, Odds ratio; *Ref*., reference; Regr. Coefficient, Regression coefficient ß.

## Data Availability

The data that support the findings of this study are available upon request from the corresponding author. The data are not publicly available due to privacy or ethical restrictions.

## Supplementary Materials

The following supporting information can be downloaded at: https://www.mdpi.com/article/10.3390/jcm12196181/s1, Table S1: Catalog of the body scanner values used in the study; Table S2: Characteristics of the overall study population (N = 73), including delivery from vertex position as well as primary cesarean section.

## Abbreviations

3D	three dimensional
CI	confidence interval
MR(I)	Magnetic resonance imaging
OC	Obstetric conjugate
OR	odds ratio
wog	weeks of gestation
