# Supplementary material for: Novel Three-Dimensional Body Scan Anthropometry versus MR-Pelvimetry for Vaginal Breech Delivery Assessment
Source: J Clin Med. 2023 Sep 25;12(19):6181. doi: 10.3390/jcm12196181 (PMC10573905; doi:10.3390/jcm12196181)
Supplement: Supplementary file 1 [file jcm-12-06181-s001.zip › jcm-2599513-supplementary.pdf]

Supplement 1

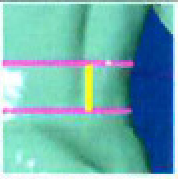	5070<br>Waist to high hip back
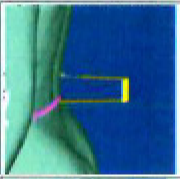	5075<br>Distance waistband-high hip back
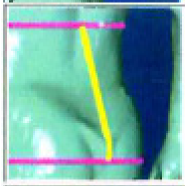	5080<br>Waist to buttock
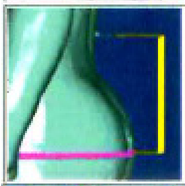	5085<br>Distance waistband - buttock
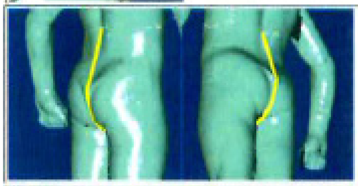	6012<br>Crotch length, rear
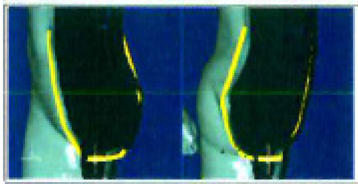	6015<br>Crotch length at waistband
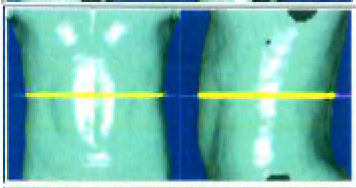	6510<br>Waist girth
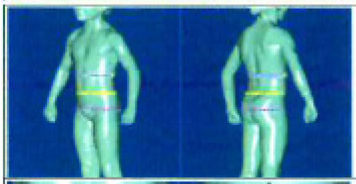	6512<br>Middle Hip
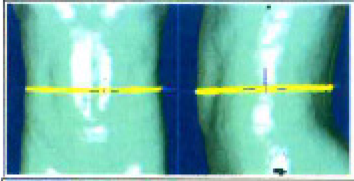	6520<br>Waist band
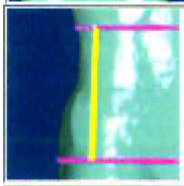	7011<br>Waist to buttock height right

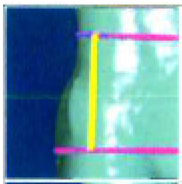	7016<br>Waistband to buttock height right
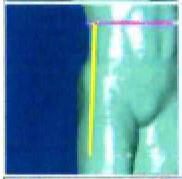	7021<br>Waist to hip/thigh right
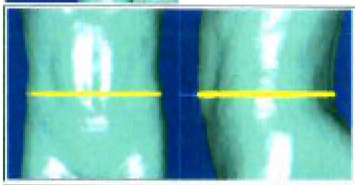	7510<br>High hip girth
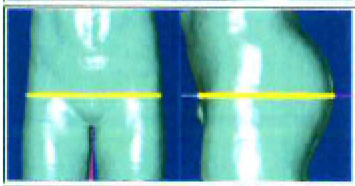	7520<br>Buttock girth
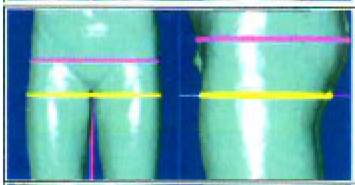	7525<br>Hip girth
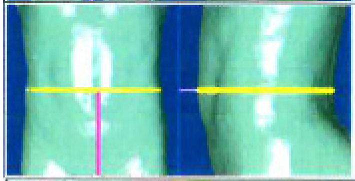	7540<br>Belly circumference
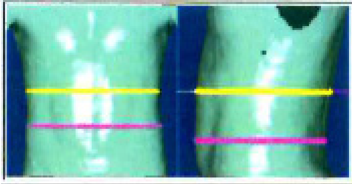	7545<br>Maximum belly circumference

Table S1. Catalog of the body scanner values used in the study.

The table shows all 17 automatically generated 3D body scanner values, used in the study. The yellow lines show the measurement planes, the purple lines show important orientation planes or boundary points when measuring distances.

Variables		N	mean	SD	95% CI	min	max
	gestational age at pelvimetry [weeks]	72	36.5	0.6	36.42 ; 36.68	35.5	39.5
	BMI [kg/m²] before pregnancy	73	22.9	3.3	22.10 ; 23.63	18.9	31.7
	BMI [kg/m²] at diagnostics	67	27.9	3.6	27.00 ; 28.75	21.9	38.6
	BMI gain [kg/m²]	67	4.9	1.7	4.48 ; 5.29	0.0	8.2
**MRI Pelvimetry**	obstetrical conjugate [cm]	72	12.8	0.9	12.56 ; 12.99	10.6	14.8
	pelvic width [cm]	72	13.5	1.0	13.29 ; 13.76	11.2	15.9
	pelvic constriction [cm]	72	11.7	1.0	11.49 ; 11.96	9.3	14.0
	sacral pelvic outlet diameter [cm]	72	13.3	0.9	13.05 ; 13.49	11.1	15.3
	coccygeal-pelvic outlet [cm]	72	8.7	1.0	8.47 ; 8.93	5.0	10.8
	interspinous distance [cm]	72	11.1	0.9	11.31 ; 11.10	8.6	13.1
	intertuberous distance [cm]	72	14.1	1.3	13.83 ; 14.45	10.3	16.9
**Pelvimeter**	external conjugate [cm]	72	23.5	2.1	23.01 ; 24.01	19.0	29.0
	distantia spinarum [cm]	72	24.1	1.8	23.65 ; 24.48	19.5	27.0
	distantia intercristarum [cm]	72	27.8	1.9	27.34 ; 28.24	24.0	36.0
	distantia trochanterica [cm]	71	33.3	2.3	32.79 ; 33.88	27.0	39.0
**3D bodyscan**	waist to high hip back (5070) [cm]	65	7.4	1.4	7.00 ; 7.72	3.7	10.5
	distance waistband to high hip back (5075) [cm]	65	4.1	2.3	3.50 ; 4.62	0.1	8.5
	waist to buttock (5080) [cm]	65	21.3	1.5	20.94 ; 21.70	17.8	24.8
	distance waistband to buttock (5085) [cm]	65	17.2	2.4	16.56 ; 17.76	12.3	21.8
	scrotch length, rear (6012) [cm]	65	42.7	2.6	42.06 ; 43.38	36.3	49.0
	scotch length at waistband (6015) [cm]	65	70.6	9.8	68.17 ; 73.03	54.2	92.4
	waist girth (6510) [cm]	65	100.9	7.5	99.03 ; 102.76	85.9	118.4
	middle hip (6512) [cm]	65	112.3	8.8	110.08 ; 114.45	95.9	135.6
	waist band (6520) [cm]	65	104.7	7.4	102.91 ; 106.58	89.4	122.7
	waist to buttock hight right (7011) [cm]	65	20.9	1.6	20.48 ; 21.26	17.4	24.5
	waistband to buttock hight right (7016) [cm]	65	14.9	3.2	14.08 ; 15.66	8.7	19.9
	waist to hip right (7021) [cm]	65	35.5	2.0	35.05 ; 36.56	30.8	40.4
	high hip girth (7510) [cm]	65	110.8	8.2	108.75 ; 112.80	94.2	134.3
	buttock girth (7520) [cm]	65	109.8	9.2	107.54 ; 112.12	92.1	134.2
	hip girth (7525) [cm]	65	110.9	9.3	108.56 ; 113.18	92.4	134.7
	belly circumference (7540) [cm]	65	109.1	7.8	107.21 ; 111.08	93.3	131.7
	maximum belly circumference (7545) [cm]	65	110.2	8.0	108.17 ; 112.14	93.7	133.4
	external conjugate [cm]	68	29.5	2.4	28.92 ; 30.07	24.7	36.2
	distantia spinarum [cm]	68	27.2	2.2	26.64 ; 27.71	22.2	32.4
	distantia intercristarum [cm]	68	34.1	2.7	33.46 ; 34.78	29.4	43.6
	distantia trochanterica [cm]	68	39.0	3.4	38.21 ; 39.88	32.0	46.2
	distance crista to trochanter (right) [cm]	68	19.1	3.8	18.15 ; 19.99	11.5	28.5
**delivery**	gestational age at delivery [weeks]	70	39.4	1.0	39.25 ; 39.63	37.2	41.4
	birth weight [g]	71	3323.2	340.3	3242.67 ; 3403.78	2690.0	4460.0
	pH value	71	7.23	0.09	7.21 ; 7.25	6.95	7.39
	APGAR score 5 minutes	71	9.3	0.9	9.07 ; 9.49	6.0	10.0

Table S2: Characteristics of the overall study population (N=73), including delivery from vertex position as well as primary cesarean section.

The numbers in brackets behind the body scanner values correspond to the description from the measurement value catalog (Supplement 1).

N, number; *BMI*, Body-Mass-Index; *SD*, standard deviation; *95% CI*, 95%-confidence interval; *min*, minimum; *max*, maximum; *5' APGAR-value*, Appearance-Pulse-Grimace-Activity-Respiration value 5minutes after delivery
